# A Single Consumption of High Amounts of the Brazil Nuts Improves Lipid Profile of Healthy Volunteers

**DOI:** 10.1155/2013/653185

**Published:** 2013-06-11

**Authors:** Elisângela Colpo, Carlos Dalton de Avila Vilanova, Luiz Gustavo Brenner Reetz, Marta Maria Medeiros Frescura Duarte, Iria Luiza Gomes Farias, Edson Irineu Muller, Aline Lima Hermes Muller, Erico Marlon Moraes Flores, Roger Wagner, João Batista Teixeira da Rocha

**Affiliations:** ^1^Department of Chemistry, Natural and Exact Sciences Centers, Federal University of Santa Maria (UFSM), 97105900 Santa Maria, RS, Brazil; ^2^Department of Nutrition, Center Franciscan University (UNIFRA), Santa Maria, RS, Brazil; ^3^Clinical Laboratory Analysis, University Hospital, Santa Maria, RS, Brazil; ^4^Lutheran University of Brazil (ULBRA), Santa Maria, RS, Brazil; ^5^Department of Technology and Food and Science, Federal University of Santa Maria (UFSM), Santa Maria, RS, Brazil

## Abstract

*Background*. This study investigates the effects of Brazil nut ingestion on serum lipid profile in healthy volunteers. *Methods*. Ten healthy subjects were enrolled in the study. Each subject was tested 4 times in a randomized crossover in relation to the ingestion of different serving sizes of the Brazil nut: 0, 5, 20, or 50 g. At each treatment point, peripheral blood was drawn before and at 1, 3, 6, 9, 24, and 48 hours and 5 and 30 days. Blood samples were tested for total cholesterol, high- and low-density lipoprotein cholesterol (HDL-c and LDL-c, resp.), triglycerides, selenium, aspartate and alanine aminotransferases, albumin, total protein, alkaline phosphatase, gamma GT, urea, creatinine, and C-reactive protein. *Results*. A significant increase of the plasma selenium levels was observed at 6 hours within the groups receiving the nuts. Serum LDL-c was significantly lower, whereas HDL-c was significantly higher 9 hours after the ingestion of 20 or 50 g of nuts. The biochemical parameters of liver and kidney function were not modified by ingestion of nuts. *Conclusions*. This study shows that the ingestion of a single serving of Brazil nut can acutely improve the serum lipid profile of healthy volunteers.

## 1. Background

Selenium is an essential nutrient for human health [1], and its biological functions are mediated by the expression of about 20 selenoproteins which have selenocysteine at their active centers [2–4]. Some selenoproteins, for example, glutathione peroxidase (GPx) and thioredoxin reductase (TrxR), are important antioxidant enzymes [3, 5–7]. However, high acute selenium ingestion can be toxic to mammals, and epidemiological observations have suggested that dietary overexposure to selenium increases the prevalence of chronic degenerative diseases such as type 2 diabetes, a myotrophic lateral sclerosis, and neoplasias [3, 8, 9].

Selenoproteins can promote cardiovascular benefits possibly via their antioxidant properties. Some isoforms of GPx are known for being able to prevent the oxidative modification of lipids (including those found in lipoproteins), inhibit platelet aggregation, and modulate inflammation by reducing the peroxide tonus [1, 3, 10–12]. Additionally, some animals as well as epidemiological studies in humans have identified a putative protective role of some GPx isoforms against cardiovascular damage [1, 13, 14]. However, some large randomized trials investigating the effects of the administration of selenium containing supplements have failed to show a significant protective effect on cardiovascular disease and mortality [14–16]. On the other hand, a meta-analysis of 25 observational studies showed a significant inverse association between selenium status with the risk of coronary heart disease (CHD), particularly within populations with low selenium intake or status [14]. A positive association between plasma selenium levels with lower atherogenic index (a reliable indicator of predisposition to heart diseases [17]) has been suggested in nutritional surveys among Japanese [18], Indians [19], and Koreans [20].

Cereals, nuts, meats, and seafood are the major sources of human dietary Se. The Se content in vegetables varies depending on several factors such as the soil in which they are grown, the Se concentration in the irrigating water, and the usage of Se-containing fertilizers [14]. Selenium concentration in Brazil nut varies between 8 and 83 *μ*g/g and is among the highest found within foods consumed by humans [21–25]. Brazil nut is also a good source of other nutrients, including unsaturated fatty acids, proteins, fiber, magnesium, phosphorus, thiamin, niacin, vitamin E, vitamin B_6_, calcium, iron, potassium, zinc, and copper. Moreover, the oily endosperm contains about 50% monounsaturated fatty acids (MUFA) [26].

As pointed out above, Se consumption and selenoenzymes (particularly GPx) have been associated with cardiovascular protection in rodents and humans [12, 14, 27, 28]. Brazil nut has a high content of selenium and could, therefore, have cardioprotective effects. In addition, different types of nuts such as peanuts, almonds, walnuts, and macadamia nuts, among others, have been shown to modulate the lipid profile in both unhealthy as well as healthy subjects [29–36]. This beneficial effect has been attributed to the high levels of MUFA and polyunsaturated fatty acids (PUFA) found in nuts [37].

Fatty acids from nuts are important contributors to the beneficial health effects which protect from the development of CHD [38]. Willett et al. [39] reported that high MUFA diets are associated with a reduced cardiovascular disease-associated mortality. Recently, a few studies have indicated a beneficial effect of long-term Brazil nut intake on serum cholesterol among obese and nonobese subjects [36, 37, 40]. However, the acute effects of the ingestion of Brazil nut on the atherogenic index of healthy subjects have not yet been evaluated. In this study, we investigate the effects of moderate to high amount Brazil nut ingestion on lipid profile, hepatic and kidney biochemical parameters in healthy volunteers to determine either beneficial or potentially toxic effect of selenium.

## 2. Methods

### 2.1. Study Subjects

Fifteen healthy subjects (8 men and 7 women) were initially recruited at the Universidade Federal de Santa Maria, Brazil. Study candidates (23–34 years old) were evaluated based on their self-reported medical history and laboratory tests. Early in the study, two male and one female subjects were excluded due to high acute alcohol intake. Two female subjects were diagnosed with hypothyroidism and were, therefore, excluded. Body weight was measured to the nearest 0.01 kg using a digital scale, and height was measured to the nearest 0.1 cm using a wall-mounted stadiometer. The body mass index (BMI) (kilograms per square meter) was calculated, and the subjects were classified according to the World Health Organization guidelines [41]. The demographics and baseline test results of the 10 selected participants (6 men and 4 women) are shown in Table 1. This study has been reviewed and approved by the Universidade Federal de Santa Maria's Internal Review Board (no. 0240.0.243.000-11), and informed consent was obtained from all participants.

### 2.2. Experimental Design

Each subject was tested 4 times following a randomized crossover regarding the administration of the different amounts of Brazil nut: 0, 5, 20, and 50 g. Two Latin squares of 4 × 4 for the 4 treatments were used to randomize participants into 4 orders of treatment. Prior to each treatment, the volunteers underwent a 30-day washout period. 

### 2.3. Brazil Nut Diet

The volunteers were given instructions by a nutritionist to exclude Se-rich foods from their diets (eggs, egg yolks, garlic, Brazil nut, whole wheat cereal, viscera, etc.) throughout the blood sampling period. 

The volunteers were given a balanced diet with daily energy requirement of 25 kcal/kg/day, a diet normocaloric. We applied 24-hour dietary recall (24 hDR) and food frequency questionnaires (FFQ) after the last blood sampling to verify the types of foods consumed during the study period. According to the United States Department of Agriculture—USDA, Brazil nut contains (per 100 g) 14.5 g of protein, 15.1 g of carbohydrates, 63.7 g of total fat (15.3 g SFA, 27.4 g MUFA, and 21 g PUFA), and 7.9 g of dietary fiber, for a total of 2,690 kJ [42].

### 2.4. Se Determination in Brazil Nut

Samples with mass up to 500 was weighed, transferred to quartz vessels together with 6 mL of concentrated nitric acid. The vessels were heated in a microwave oven with maximum temperature and pressure of 280°C and 80 bar, respectively.

### 2.5. Lipid Determination in Brazil Nut

The extraction of Brazil nut was performed according to the method described by Bligh and Dyer [43], grinding a known amount of Brazil nuts in the presence of a methanol/chloroform (1 : 2 v/v) mixture at 30 mL/g of fresh weight. The fatty acid methyl esters were analyzed by a gas chromatograph using a procedure described by Christie [44]. The results were expressed as relative percent of total fatty acids according to Visentainer [45].

### 2.6. Blood Samples Collection

Blood samples were collected by venous puncture prior to and at 1, 3, 6, 9, 24, and 48 hours and 5 and 30 days after the ingestion of nuts. Except for the 6- and 9-hour time points, all volunteers were at a 12-hour fasting period for the collection of blood. Blood samples were collected by venous puncture into Vacutainer (BD Diagnostics, Plymouth, UK) tubes with no anticoagulant and EDTA anticoagulant. Blood samples stored in ice were routinely centrifuged within 1 h after collection at 2500 ×g for 15 min. Aliquots of serum samples were immediately used to assess fasting glucose, total cholesterol (TC), high-density lipoprotein cholesterol (HDL-c), triglycerides, aspartate and alanine aminotransferases (AST and ALT, resp.), albumin, total protein, alkaline phosphatase, gamma GT, urea, creatinine, and C-reactive protein (CRP). Aliquots of plasma were used for selenium measurements. Serum and plasma samples were then stored at −80°C for up to 4 weeks before the analyses.

### 2.7. Blood Tests

Hemoglobin levels and hematocrit were determined in a Cobas Micros system (Hematology Analyzer, Roche Diagnostics). Fasting glucose, TC, HDL-c, triglycerides, AST, ALT, albumin, total protein, alkaline phosphatase, gamma-GT, urea, creatinine, and CRP measurements were performed using Ortho-Clinical Diagnostics reagents on a fully automated analyzer (Vitros 950 dry chemistry system, Johnson & Johnson, Rochester, NY, USA). Low-density lipoprotein cholesterol (LDL-c) was calculated using the Friedewald equation [46].

### 2.8. Atherogenic Index (AI) Determination

The atherogenic index was calculated as the ratio between total cholesterol and HDL-c or as the ratio between LDL-c and HDL-c concentrations according to Kinosian et al. [47] and Lemieux et al. [48]. 

### 2.9. Se Concentration in Plasma

The plasma Se concentration was determined using atomic absorption spectrometry with graphite furnace atomizer (GFAAS) and Zeeman Effect background correction. Samples were diluted with Triton X-100. Palladium chemical modifier, wavelength 196.0 nm, pyrolysis temperature 140°C, and atomization temperature 220°C were used.

### 2.10. Statistical Analysis

Data are expressed as mean ± Standard Deviation (SD). The statistical analysis was performed using analysis of variance with measure repeated (ANOVA) and nonparametric tests (Wilcoxon). Descriptive statistics was performed for all baseline characteristics. Differences were considered significant when *P* < 0.05.

## 3. Results

The volunteers included in the study were 24.7 ± 3.4 years old (range 23–34 years old). Demographic, anthropometric, and laboratory characteristics are listed in Table 1.

The average Se concentration in Brazil nut was 31.25 ± 18.7 *μ*g/g. Therefore, the net Se intake was about 156 *μ*g, 625 *μ*g, and 1560 *μ*g for the groups ingesting 5, 20, and 50 g of nuts, respectively. The estimated fat intake from nuts is shown in Table 2.

The biochemical parameters of liver and kidney function in healthy volunteers, such as AST, ALT, alkaline phosphatase, Gama GT, urea, and creatinine, were not modified by ingestion of nuts, indicating an absence of hepatic and renal toxicity of high amounts of Brazil nuts intake. PCR was also evaluated, and there was no change in its levels after ingestion of Brazil nuts (data not shown).

**Figure 1:**
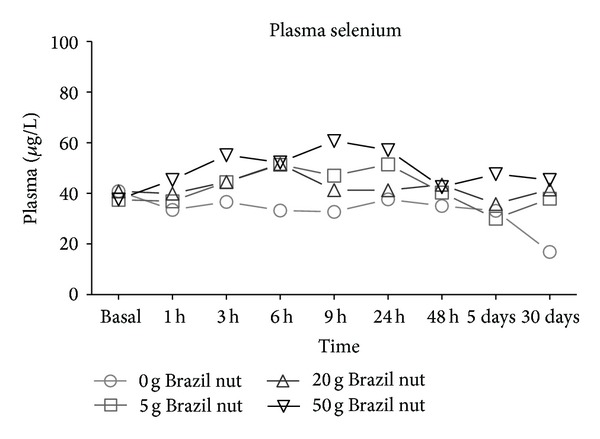
Plasma levels of selenium in healthy volunteers after consumption of the Brazil nut. Measures repeated—ANOVA and Wilcoxon tests.

Plasma selenium levels were significantly increased in all groups 6 hours after the ingestion of Brazil nut. Moreover, at the highest dose (50 g) the Se increase was evident starting at as early as 3 h and remained above baseline levels for up to 24 h (Figure 1). At 48 hours, the plasma Se levels did not differ from its baseline concentration (Figure 1).

**Figure 2:**
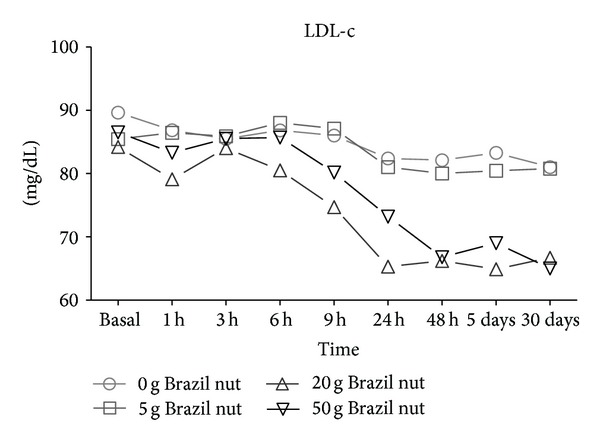
Seric levels of LDL-c in healthy volunteers after consumption of the Brazil nut. Measure repeated—ANOVA and Wilcoxon tests.

**Figure 3:**
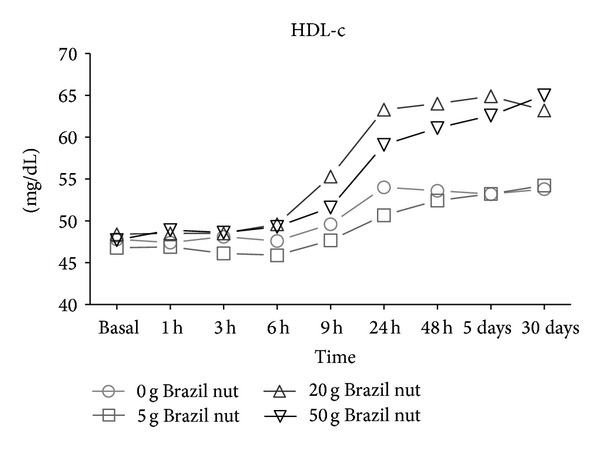
Seric levels of HDL-c in healthy volunteers after consumption of the Brazil nut. Measure repeated—ANOVA and Wilcoxon tests.

Serum LDL-c levels were significantly lower starting at 9 hours after the ingestion of nuts within the groups receiving 20 or 50 g and reached a steady level at 48 hours (Figure 2). Subjects that consumed higher amounts of Brazil nut exhibited an increase in HDL-c starting at 6 hours after the intake which reached a stable level at 5 days (Figure 3, *P* < 0.05). 

Interestingly, the ingestion of 20 g of Brazil nut determined a more pronounced decrease in LDL-c levels as well as a higher increase in HDL-c than did 50 g. These results suggest that eating an average of 4 nuts might be enough to improve the levels of LDL-c and HDL-c for up to 30 days.

Accordingly, the AI (TC/HDL-c and LDL-c/HDL-c ratio) was decreased in subjects that consumed 20 and 50 g of Brazil nut (*P* < 0.05, data not shown). Serum triglycerides and total cholesterol did not significantly vary (*P* > 0.05) within the study time frame (data not shown).

Even though the measured plasma Se concentrations did not significantly vary following the ingestion of 5, 20, or 50 g of nuts, changes in LDL-c and HDL-c were only observed with the ingestion of 20 or 50 g which persisted for up to 30 days. These results raise the question of whether the beneficial effects of Brazil nut on the atherogenic index may be due to factors other than selenium (MUFA and PUFA perhaps), highlighting the importance of studying the separate and combined effect of selenium and fatty acids on atherogenic indexes.

## 4. Discussion

Regular nut intake has been associated with many health benefits in adults [37, 49, 50]. The results of this study support the notion that the consumption of a single serving of nuts can acutely beneficially modify serum lipids.

Contrasting with diets rich in SFA, MUFA- and PUFA-rich foods are potentially beneficial for health [38]. Nuts are generally low in saturated fatty acids and high in unsaturated fatty acids [51]. Unsaturated fatty acids (both mono- and polyunsaturated) have been shown to reduce serum TC and LDL-c. Brazil nut is a good source of unsaturated fat (~50% MUFA). However, despite knowing that the Brazil nut has high concentrations of unsaturated fatty acids when compared with other nuts such as macadamias, almonds, walnuts, pecans, pistachios, and peanuts, the Brazil nut has a relatively higher content of SFAs. 

Therefore, the increase in HDL-c observed in this present study may be attributed to the higher MUFA and SFA content in Brazil nut [52]. According to Riccardi et al. [53], SFA and MUFA increase HDL-c, whereas high intakes of PUFA decrease HDL-c. Unsaturated fatty acids have been shown to increase HDL-c less than SFAs do [54]. Furthermore, while the unsaturated fatty acid profile of nuts (high MUFA and PUFA) is thought to mediate the majority of the beneficial effects of nuts on serum lipids, other components such as fiber and selenium might contribute to these effects [14, 26, 31, 55].

Many studies have shown that chronic intake of varying amounts of nuts is effective to increase the blood concentrations of Se and improve lipid profile [29–37, 40, 56–59]. A meta-analysis by Flores-Mateo et al. [14] based on several observational studies pointed to an inverse correlation between plasma selenium concentrations and coronary heart disease incidence. But the validity of such correlations needs further confirmation. Stranges et al. [60] concluded that an increase in plasma selenium in adult population was associated with increased total and non-HDL cholesterol levels but not with HDL-c. Moreover, evidence showing that low selenium status is a cardiovascular risk factor must still be considered provisional.

In conclusion, the results obtained here suggest that the consumption of a single serving of Brazil nut is sufficient to improve the lipid profile of healthy volunteers (lowered LDL-c and raised HDL-c), without producing hepatic and renal toxicity of high amounts of Brazil nuts intake. However, further investigation is needed to validate the beneficial effects of Brazil nut because here we have used a small number of subjects. In addition, it is also important to evaluate the isolated and combined effects of selenium and/or unsaturated fatty acids found in Brazil nuts on atherogenic parameters in order to better understand their mechanistic role in modulating cardiac indexes in healthy and dyslipidemic subjects. In addition, the evaluation of the effects of chronic consumption of Brazil nuts and the inclusion of dyslipidemic patients are paths to be followed.

## Figures and Tables

**Table 1:** Anthropometric and biochemical variables baselines of subjects.

Characteristic	Men (*n* = 6)	Women (*n* = 4)	Reference
Weight (Kg)	87 ± 13.8	59.5 ± 6.6	
BMI (Kg/m^2^)	26.9 ± 3.9	23.4 ± 1.6	18.5–24.9
Plasma selenium (*µ*g·L^−1^)	42.4 ± 16.6	36.6 ± 12.3	53 ± 20.7–161 ± 19
Leucocytes (10^3^/mm^3^)	7.2 ± 1.4	7.4 ± 1.7	3.6–11
Hematocrit (%)	44.4 ± 1.6	38.8 ± 2.8	§: 39–53
			∣: 36–48
Hemoglobin (g/dL)	14.8 ± 0.6	12.8 ± 1	§: 12.8–17.8
			∣: 11.6–15.6
Fasting glucose (mg/dL)	82.4 ± 7.8	82.9 ± 7.4	70–99
Albumin (g/dL)	4.7 ± 0.4	4.8 ± 0.4	3.5–5.5
Total protein (g/dL)	6.5 ± 0.6	6.9 ± 0.3	6–8
Cholesterol total (mg/dL)	145 ± 4.8	143 ± 6.7	<200
HDL-c (mg/dL)	47.6 ± 2.3	47.8 ± 2.5	≥35
LDL-c (mg/dL)	87.7 ± 9.8	84.5 ± 6.5	<130
Triglycerides (mg/dL)	60.5 ± 16.9	53.3 ± 16	<150

Results are expressed as mean ± S.D. §: masculine; ∣: females.

**Table 2:** Fatty acids composition of the Brazil nut.

Fatty acids	g/100 g	SD
AGS		
C14:0	0.03	0.00
C16:0	10.49	0.31
C17:0	0.09	0.04
C18:0	8.52	0.26
C22:0	0.04	0.00

Total	19.16	0.12

MUFA		
C16:1	0.28	0.02
C18:1n9 cis	28.61	1.52
C20:1	0.05	0.00

Total	28.94	0.51

PUFA		
C18:2n6 cis	26.25	1.07
C18:3n6	0.24	0.01
C18:3n3	0.06	0.01

Total	26.55	0.36

MUFA: monounsaturated fatty acids; PUFA: polyunsaturated fatty acids; SFA: saturated fatty acids.

## Abbreviations

GPx: Glutathione peroxidase
TrxR: Thioredoxin reductase
CHD: Coronary heart disease
MUFA: Monounsaturated fatty acids
PUFA: Polyunsaturated fatty acids
SFA: Saturated fatty acids
BMI: Body mass index
24 hDR: 24-hour dietary recall (24 hDR)
FFQ: Food frequency questionnaires
TC: Total cholesterol
HDL-c: High-density lipoprotein cholesterol
LDL-c: Low-density lipoprotein cholesterol
AI: Atherogenic index
AST: Aspartate aminotransferases
ALT: Alanine aminotransferases
CRP: C-reactive protein.
